# Factors associated with dementia-related stigma in British adolescents

**DOI:** 10.1186/s12889-024-20419-7

**Published:** 2024-10-21

**Authors:** Esra Hassan, Ben Hicks, Naji Tabet, Nicolas Farina

**Affiliations:** 1 Centre for Dementia Studies, Brighton and Sussex Medical School, Trafford Centre, University of Sussex, Brighton, BN1 9RX UK; 2 Peninsula Medical School, University of Plymouth, Plymouth Institute of Health and Care Research, Plymouth, Devon PL4 8AA UK

**Keywords:** SEM, Dementia attitudes, Students, Ageism, Adolescents, Stigma

## Abstract

**Background:**

Dementia-related stigma is a prominent barrier for people living with dementia, leading to poor well-being and social isolation. Adolescents are an under-researched group in society that may already have experience of dementia and are more susceptible to attitudinal change which makes them ideal targets for anti-stigma initiatives outlined by public health policy. For the development of evidence-based anti-stigma initiatives in adolescents, it is important to understand which socio-demographic groups are most likely to develop stigmatising attitudes and why. This study aims to identify factors of dementia-related stigma in adolescents.

**Methods:**

A total of 1,044 adolescents (aged 11–18 years) from across six regions of England were included in the analysis of this cross-sectional, survey-based study. Descriptive statistics and multiple regressions were employed to explore the association between demographic variables, modifiable factors of dementia-related stigma and the outcome of dementia-related stigma. A path analysis via a structural equation model was employed to test for direct and mediatory effects.

**Results:**

Multiple regression models revealed that younger adolescents, those with higher levels of contact with dementia, higher levels of empathy, higher levels of dementia knowledge, and higher affinity to older adults, are associated with more positive dementia attitudes in adolescents (*p* < 0.05). Within the accepted structural equation model, empathy, level of contact and dementia knowledge were key mediators of dementia-related stigma (*p* < 0.05).

**Conclusion:**

This study highlights that modifiable factors such as level of contact, ageism, and empathy have a potentially important role in how dementia-related stigma may start to form in the adolescent years. Developing contact-based strategies that stimulate empathetic responses may be useful targets for stigma reduction initiatives for adolescents.

**Supplementary Information:**

The online version contains supplementary material available at 10.1186/s12889-024-20419-7.

## Background

Dementia affects an estimated 55.2 million individuals worldwide [1]. With a globally ageing population, it is predicted that the number of people living with dementia will also grow. One persistent challenge faced by people living with dementia is the stigma attached to the condition, which creates barriers to accessing support and impacts the quality of life and well-being of those living with dementia and their families [2]. Such discrimination is commonplace, with 83% of people with dementia surveyed reporting discrimination in one or more areas of life [3]. Both national and international policies recognise and emphasise the need to combat dementia-related stigma as a public health policy priority [4]. Public education about dementia and anti-stigma initiatives are widely accepted strategies to achieve this [5–7].

Dementia-related stigma captures the essence of ‘public stigma’ [8]. Stigma is defined as a collection of negative attitudes and beliefs that lead to discriminatory behaviour towards a particular group (i.e., people living with dementia) [9]. Public stigma includes interrelated terms such as ‘attitudes’, ‘stereotypes’, ‘perceptions’, ‘beliefs’, and ‘discrimination’ [10]. Like much of the literature, theoretical frameworks tend to be developed with mental illness in mind and then applied to dementia [11].

Adolescents represent part of the wider society and there is a growing number of grandchildren of people living with dementia [12]. Previous research has established that adolescents already exhibit some negative attitudes towards dementia [13]. Adolescents are considered ideal targets for anti-stigma initiatives due to the ‘impressionable year’s hypothesis’. The impressionable year's hypothesis outlines that adolescence is a key developmental stage that plays a major role in shaping attitudes. Relationships and social networks are established during this developmental period that can contribute to their perceptions and worldviews [14]. Despite these findings, existing research has mostly focused on adults above 18 years old, including many stigma-reduction initiatives [15]. Where initiatives like dementia awareness programmes have been implemented among under-18-year-olds [5, 16], the heterogeneity across the studies limits the interpretation of their efficacy in reducing dementia stigma. Some of the research findings highlight no benefit over control groups [17]. To maximise the likelihood of developing a successful stigma reduction intervention, researchers must establish the factors that may be associated with dementia-related stigma. In adults, factors such as limited dementia knowledge, being male, and lack of contact with dementia are associated with poorer attitudes [18–20]. There is a lack of consensus on the factors determining dementia-related stigma among adolescents due to the lack of literature in this area [21]. For example, empathy is an important construct within the broader adult dementia stigma literature [22, 23], yet only one qualitative [24] and one quantitative paper [25] have explored this relationship in adolescents. Conceptually, empathy is multi-faceted and includes various constructs (e.g., affective, prosocial, and cognitive) that can be measured. Thus, one study could be tapping into a single component of empathy, while another could be broadly tapping into several components (e.g., contagion and empathetic concern versus compassion). Given the multitude of concepts, methodological approaches differ accordingly [26].

Recent evidence from a homogenous sample (predominately White British adolescents from a single region of England) indicates the complex relationships between dementia-related stigma and factors such as gender and empathy [25]. This study aims to build upon this evidence by recruiting a more diverse sample (via the inclusion of more geographic locations of England, religious groups, and ethnic groups) of adolescents to better understand which socio-demographic groups might be more likely to hold stigmatising attitudes. In addition, this study differs from other studies (e.g., [13, 25]) by incorporating more outcomes from the broader literature (e.g., ageist beliefs) and analysing them through exploratory structural equation modelling (SEM). Prior studies have not yet explored factors such as ageism and level of dementia knowledge in the context of dementia-related stigma in adolescents using SEM.

The exploratory SEM incorporates elements from three main frameworks. This includes 1) the empathy-altruism hypothesis [27] in which empathy towards a stigmatised group may change attitudes [28], 2) the contact hypothesis [29] in which increased intergroup contact can lead to reduced prejudice and improved attitudes towards a stigmatised group [30], and 3) the attribution model of stigma in which attributions can impact the stigmatising attitudes, emotional reactions, and behaviours they hold towards people living with dementia [31]. These frameworks were chosen as they explore the role of empathy as a potential mediator between contact and dementia-related stigma [25].

## Methods

### Study design

This paper presents baseline quantitative data collected as part of a quasi-longitudinal study. The study consisted of adolescents aged 11–18 years (reflecting the ages of secondary education pupils in England) from secondary schools across England completing a series of questionnaires relating to demographic information and topics concerning dementia-related stigma. The survey was distributed between February 2023 and April 2023.

### Study setting and sample

The inclusion criteria for participants were a) being aged 11–18 years old, b) attending a mainstream secondary school/sixth-form setting in England (in which a gatekeeper such as the headteacher has given consent for the school to take part in the study), and c) having obtained parental consent (opt-in/opt-out) to be part of the study. The exclusion criteria consisted of adolescents, a) within a non-mainstream school (i.e., special educational needs schools) and b) who do not have the capacity to consent (determined by the school).

Schools were initially contacted, via email, where public contact information existed. Of the 305 schools contacted, 273 schools did not respond to the invitation, while 21 schools abstained from participation. Reasons for refusal given were lack of capacity to support the study due to staff shortages, and time pressures resulting from a tight curriculum. Eleven schools across six regions of England consented to taking part in the study. However, two of the schools withdrew after consenting. The schools did not provide any reasons for their withdrawal. Thus, nine schools in total took part in the study. In total, 1,625 adolescents aged 11–18 years old were approached, of which, 1,453 provided written consent to take part. No reasons for refusal were given by participants.

### Public Patient Involvement (PPI) and pilot testing

Two groups were consulted in the design of the study: 1) Individuals with dementia experience (*n* = 6) and, 2) adolescents (*n* = 3). In part, these groups were used to guide decisions about outcome choices. As a result of the consultation, additional signposted resources were added to the debrief form for participants if they wanted to find out more about dementia. The panel were compensated (in monetary value) for their time.

### Ethical considerations

The study was approved (ER/BSMS9PCH/1) by the Brighton and Sussex Medical School Research Governance and Ethics Committee. School headteachers and other senior school staff were consulted for their expertise in conducting such a study within the school context. The researcher obtained an enhanced Disclosure and Barring Service (DBS) certificate. Informed consent from a parent or legal guardian for study participation was obtained.

### Procedure

Schools gatekeepers were identified and were sent an email invitation to take part in the study and were provided with a brief study overview, a detailed study information sheet and consent procedures. The gatekeeper was asked for written informed consent and facilitated the distribution of the survey. Schools had the option to distribute the survey online (via Qualtrics) or by pen and paper.

Gatekeepers sent out opt-in and opt-out consent and information sheets to parental guardians. Young people under 13 years old were required to have opt-in consent, whilst over 13-year olds required opt-out consent.. The questionnaire link (or paper copy version) was disseminated to participants a week before the data collection date. On the day of the study, all participants were required to complete a consent form before participation if they wished to take part. The questionnaires were self-completion and took approximately 10 min to complete. At the end of the questionnaire, participants were presented with a debrief form and entered into a prize draw to win a voucher.

### Variables

#### Independent variables

- *Demographic variables:* age, sex, ethnicity, and religion.
- *Heard of dementia:* A single item question whether they had heard of the term’s dementia or Alzheimer’s disease before [5]. The response choices were ‘I have heard of both dementia and Alzheimer’s disease’, ‘I have only heard of Alzheimer’s disease’, ‘I have only heard of dementia’, and ‘I have never heard of either terms’.
- The Adolescent Level of Contact with Dementia (ALOCD) questionnaire is a validated measure (α = 0.86 within the present sample) of the level of contact adolescents have with dementia [32]. The scale consists of 10 items measuring indirect (e.g. ‘I have come across adverts about dementia’) and direct contact (e.g. ‘I have come across people living with dementia’) and is rated on a 5-point Likert scale that ranges from ‘1 – Never’ to ‘5 – A great deal’. Higher scores indicate a greater frequency of dementia contact.
- The Northern Ireland Life and Times Survey (NILTS) [33] is a measure that captures a national representation of social attitudes. A module of the survey is ‘Knowledge of dementia’. This consists of seven true or false statements related to knowledge of dementia. Participants select either ‘True’, ‘False’ or ‘Don’t Know’ [34]. Higher levels of dementia knowledge denote more correctly answered statements in the NILTS.
- The EmQue-CA [35] is a validated measure of adolescent empathy that consists of components, ‘affective empathy’, ‘cognitive empathy’, and ‘intention to comfort’. Affective empathy refers to the ability to share and feel the emotions of others. Cognitive empathy is the capacity to understand someone else’s emotional state, and the intention to comfort captures the motivation to help (prosocial) [35]. The measure consists of 18 items (i.e., “If a friend is sad, I also feel sad”) with each item on a 3-point Likert response scale that ranges between ‘not true’, ‘sometimes true’, and ‘often true’ [36]. The measure had good internal reliability (α = 0.88 within the present sample). Higher total scores indicate a higher level of empathy.
- The ‘collective affinity for older people’ subscale of the Relational Ageism Scale (RAS) [37] is a 5-item measure of ageism. Each item is on a 5-point Likert scale with a response scale ranging between ‘1 – strongly agree’ to ‘5 – strongly disagree’. The collective affinity for older people subscale is most in line with public stigma frameworks. Higher scores indicate higher levels of ageism.

### Dependent variables

- The Brief version of the Adolescent Attitudes towards Dementia Scale (Brief A-ADS) is a validated measure consisting of 13 items [38] taken from the 23-item A-ADS [39]. The measure demonstrated very good internal consistency within the present sample (α = 0.86). Each item (i.e., “people with dementia can be creative”) is on a 5-point Likert scale with the response scale ranging between ‘1 – strongly disagree’ and ‘5 – strongly agree’. Higher scores indicate more positive attitudes towards dementia.
- Attribution questionnaire children’s version (AQ-8-C) is a shorter, modified eight-item children’s version of the attribution questionnaire of public stigma towards mental illness [40] designed for ages 10–18 years old [41]. The AQ-8-C represents the attribution constructs (i.e., beliefs, emotional, and behavioural) on a nine-point Likert scale ranging from 1 (not at all) to 9 (very much). Higher scores indicate fewer stigmatising attitudes. Participants responded to the items about a vignette that was adapted from the AQ-8-C where ‘mental illness’ was replaced with ‘dementia’. See supplementary material A for the vignette. The attribution questionnaire shows a generally acceptable internal and test–retest reliability in college age students (α = 0.55 to α = 0.87) [42]. In this present study the internal reliability of the AQ-8-C was α = 0.66.

### Data analysis

Participants who had never heard of dementia or Alzheimer’s before (*n* = 83) were excluded from the study to remove those who had not formed any opinions about the condition. Insufficient error responding (IER) was handled using the maximum Longstring index [43]. This is where the maximum number of consecutive values were calculated for items within the Brief A-ADS. All cases two standard deviations above the mean were excluded from the analysis (m = 6.94 ± SD. = 2.66) (*n* = 85 excluded) [44].

Standardised scoring was applied to the Brief A-ADS, ALOCD, EmQue-Ca, AQ-8-C, BSDS, and the RAS. For the NILTS, total correct scores were calculated for each participant with an overall mean percentage calculated across all scores. A missing values analysis was used to detect whether data were missing at random (*p* > 0.05). Multiple imputation (MI) was used to handle missing data and was selected to retain the statistical power of the sample size [45]. Before running the MI, cases with substantial missing values (over 50% missing) were not included in the analysis. Descriptive statistics, checks for normality and cross-tabulations were obtained. Categorical variables were recoded into dummy variables (e.g., White British = 1, other ethnic background = 0).

A multivariate linear regression was used to assess what variables (age, sex, contact, empathy, ethnicity, religion, knowledge, and whether participants have heard of dementia or Alzheimer’s disease before) were associated with dementia-related stigma outcomes. The variables were entered simultaneously into the models with the Brief A-ADS or AQ-8-C as the dependent variable. A *priori* sample size was calculated. Eight hundred and fifty participants were needed to detect a small effect size (*f*^2^ = 0.02) in a model with 11 variables (beta = 0.80, alpha = 0.05). Assumptions to run the regressions were met (Durbin-Watson statistic values were all between 1.5 and 2.5, and plots and multicollinearity were checked with the VIF values less than ten). An alpha of 0.05 was used to denote statistical significance. Validity and internal consistency checks were undertaken and are reported in supplementary material B. A bivariate Spearman’s Rho was used to assess the association between the dependent variables; the Brief A-ADS and AQ-8-C. SPSS (version 28) (IBM, New York, USA) was used to analyse the data. A statistician was consulted on the planned data analysis.

To build an exploratory model to determine model fit [46], a recursive path analysis via SEM using the maximum likelihood estimation (CB-SEM) was used to explore direct and indirect (mediation) effects between factorsData for the SEM was analysed on the IBM SPSS Amos Graphics (version 28) (IBM, New York, USA). The SEM had four stages which included testing a measurement model, model identification to assess initial model fit, model fitting following modifications and checking for direct effects, and lastly, obtaining specific indirect effects by creating specific parameter paths. The factors included in the model building were decided by the factors that were the strongest independent variables in the regressions as well as the wider theorised relationships in the literature.

For the preparation of the SEM measurement model, the AQ-8-C underwent scale reversal and factor reduction using principal component analysis. This was because higher AQ-8-C scores represent more stigmatising attitudes while higher scores on the Brief A-ADS represent fewer stigmatising attitudes. To create a latent variable where both variables score in the same direction and correlate, the AQ-8-C was summed and underwent scale reversal so that higher scores equated to fewer stigmatising attitudes. See supplementary material C for further methodological details relating to the measurement model.

A bootstrapping procedure (100,000 samples) was applied due to having non-normal data [47]. Unstandardised coefficient betas, standardised coefficients, standard error (s.e.) and bias-corrected CI (100,000 bootstrap samples at 95%) are reported for the direct and indirect effects for the CB-SEM [48] and to obtain more accurate estimations of the lower and upper limits of the CI [49]. See supplementary material D for further SEM methodology details. A Bollen-Stine bootstrap was also utilised to obtain a goodness-of-fit statistic to provide the model fit (*p-*value above 0.05 indicates good fit) [50]. Supplementary material E outlines the indexes used as indicators for the model fit [51].

## Results

There were 1,625 participants recruited. Following the data-cleaning procedure, 1,044 students remained in the analysis. On average, participants were 14.2 years old (SD = 1.00) and consisted of roughly an equal number of females (50.6%, *n* = 528) and males (49.4%, *n* = 516). The majority of participants were White British, 58.7% (*n* = 613), identified as having no religion (52%), and resided within the Bedfordshire region (52.8%, *n* = 551). Table 1 outlines the descriptive statistics obtained.

**Table 1:** Descriptive statistics (*n* = 1044)

Variables	Categories	N (valid %)	M ± SD
**Region**	Bedfordshire	551 (52.8%)	
	Leicester	76 (7.3%)	
	Brighton and Hove	257 (24.6%)	
	Hertfordshire	69 (6.6%)	
	Plymouth	78 (7.5%)	
	Birmingham	13 (1.2%)	
**School**	School 1	375 (35.9%)	
	School 2	138 (13.2%)	
	School 3	38 (3.6%)	
	School 4	95 (9.1%)	
	School 5	69 (6.6%)	
	School 6	78 (7.5%)	
	School 7	162 (15.5%)	
	School 8	76 (7.3%)	
	School 9	13 (1.2%)	
**Sex**	Female (0)	528 (50.6%)	
	Male (1)	516 (49.4%)	
**Age**			14.32 ± 1.00
	11	7 (0.7%)	
	12	8 (0.8%)	
	13	204 (19.5%)	
	14	397 (36.3%)	
	15	316 (30.3%)	
	16	127 (12.2%)	
	17	2 (0.2%)	
	18	1 (0.1%)	
**Ethnicity (dichotomised)**	White (1)	613 (58.7%)	
	All other ethnic background (0):	431 (41.3%)	
	Black South Asian East Asian Middle Eastern Mixed or multiple ethnic background Other ‘I do not want to answer’	46 (4.4%) 235 (22.5%) 20 (1.9%) 14 (1.3%) 77 (7.4%) 18 (1.7%) 21 (2.1%)	
**Religion (dichotomised)**	No religion (1)	52%	
	All other religions (0):	48%	
	Christian Buddhist Hindu Jewish Muslim Sikh Other religion ‘I do not want to answer’	199 (19.1%) 3 (0.3%) 89 (8.5%) 5 (0.5%) 130 (12.5%) 15 (1.4%) 5 (0.5%) 55 (5.2%)	
**Whether participants have heard of either Alzheimer’s Disease or Dementia (dichotomised)**	I have heard of only one of these terms (0)	223 (21.4%)	
	I have heard of both terms (1)	821 (78.6%)	
**Empathy: the empathy questionnaire for children and adolescents**	EmQue-CA total		23.42 ± 6.41
**Contact: adolescent level of contact towards dementia scale**	ALOCD total		19.90 ± 7.56
**Ageism: the relational ageism scale**	RAS total		11.10 ± 3.76
**Knowledge: Northern Irelands life and times survey**	NILTS total correct %		53% (0.53 ± 0.21)
	Low knowledge (below 50%)	453 (43.4%)	
	Good knowledge (above 50%)	591 (56.6%)	
**Stigma: attribution questionnaire 8 items – children’s version**	AQ-8-C total		28.07 ± 8.32
	AQ-8-C Log^10^ transformed total		1.43 ± 0.12
**Brief adolescent attitudes towards dementia scale**	Brief A-ADS total		48.2 ± 6.67

### The agreement between the AQ-8-C and the Brief A-ADS

The AQ-8-C and Brief A-ADS demonstrated a weak negative correlation (rs = -0.13, *p* < 0.001). 

### Regression models: Brief A-ADS

The multiple linear regression of all independent variables (k = 9) significantly fitted to the model where the Brief A-ADS was the outcome, *F*(9, 1034) = 51.50, *p* < 0.001. A significant amount of variance in the Brief A-ADS scores was explained by the model fit, *R*^2^ adjusted = 0.30. Within the model, age (*p* = 0.03) and ageist beliefs (*p* < 0.001) were negatively associated with Brief A-ADS. Having higher levels of dementia knowledge (*p* < 0.001), higher levels of dementia contact (*p* < 0.001), and higher levels of empathy (*p* < 0.001) was positively associated with Brief A-ADS. Table 2 provides the multivariate linear regression for the Brief A-ADS and the AQ-8-C.

**Table 2:** Multivariate linear regression for variables associated with the Brief A-ADS and the AQ-8-C scores (*n* = 1034)

Independent variables	Brief A-ADS (*p* < .001)				AQ-8-C (*p* < .001)			
	**β**	**B**	***p***	**CI [95%]**	**β**	**B**	***p***	**CI [95%]**
**Sex: Male**	-.03	-.43	.24	-1.15 – .29	-.02	-.01	.62	-.02 – .01
**Age**	-.06	-.38	.03*	-.72 – -.03	.06	.01	.03*	.00 – .02
**Contact**	.21	.35	< .001***	.26 – .44	.01	.00	.82	-.00 – .00
**Empathy**	.23	.24	< .001***	.19 – .30	-.27	-.01	< .001***	-.01 – -.01
**Ageism**	-.33	-.58	< .001***	-.68 – -.49	.31	.01	< .001***	.01 – .02
**Knowledge**	.13	4.06	< .001***	2.32 – 5.80	.03	.03	.28	-.02 – .07
**Ethnicity: White**	.02	.27	.53	-.57 – 1.11	-.05	-.02	.15	-.04 – .01
**Religion: No religion**	-.06	-.80	.06	-.62 – .02	.04	.01	.29	-.01 – .03
**Heard of dementia or Alzheimer's: heard of both terms**	-.05	-.86	.06	-1.70 – .03	-.03	-.01	.24	-.04 – -.02
**Multiple linear regression** **(*n* = 1034)**	**R**	***R***^**2**^	**Adjusted *R***^**2**^	**F**	**R**	***R***^**2**^	**Adjusted R**^**2**^	**F**
	.56	.31	.30	51.50	.44	.20	.19	28.12

Dichotomous variables are coded as 0 versus 1 (1 denotes the comparator)

*B* unstandardised beta coefficient, *β* standardised beta coefficient, *CI* confidence interval (lower – upper bound) [95%]. Regression model summary indicated by R, *R*^2^, adjusted *R*^2^, and F values for Brief A-ADS and AQ-8-C

Outcome variable: Brief A-ADS and AQ-8-C. Statistical significance (*p*): * = *p* < 0.05; *** *p* =  < 0.001

### Regression models: AQ-8-C

The multiple linear regression of all independent variables (k = 9) significantly fitted to the model where the AQ-8-C was the outcome, *F*(9, 1034) = 28.12, *p* < 0.001. A small but significant amount of variance in the AQ-8-C scores was explained by the model fit, *R*^2^ adjusted = 0.19. Within the model, age (*p* = 0.03) and ageist beliefs (*p* < 0.001) were positively associated with AQ-8-C. Lower levels of empathy were associated with higher levels of stigma towards dementia (*p* < 0.001). Table 2 provides the multivariate linear regression for the Brief A-ADS and the AQ-8-C.

### Structural equation model

Due to their significance in the regression models, the following independent variables were taken forward to the SEM: age, contact, empathy, ageism, and knowledge. Sex was also added to the model due to prior empirical evidence of the role of sex on attitudes through mediatory mechanisms [23]. The measurement SEM and SEM methodology are reported in supplementary materials C and D.

**Fig. 1:**
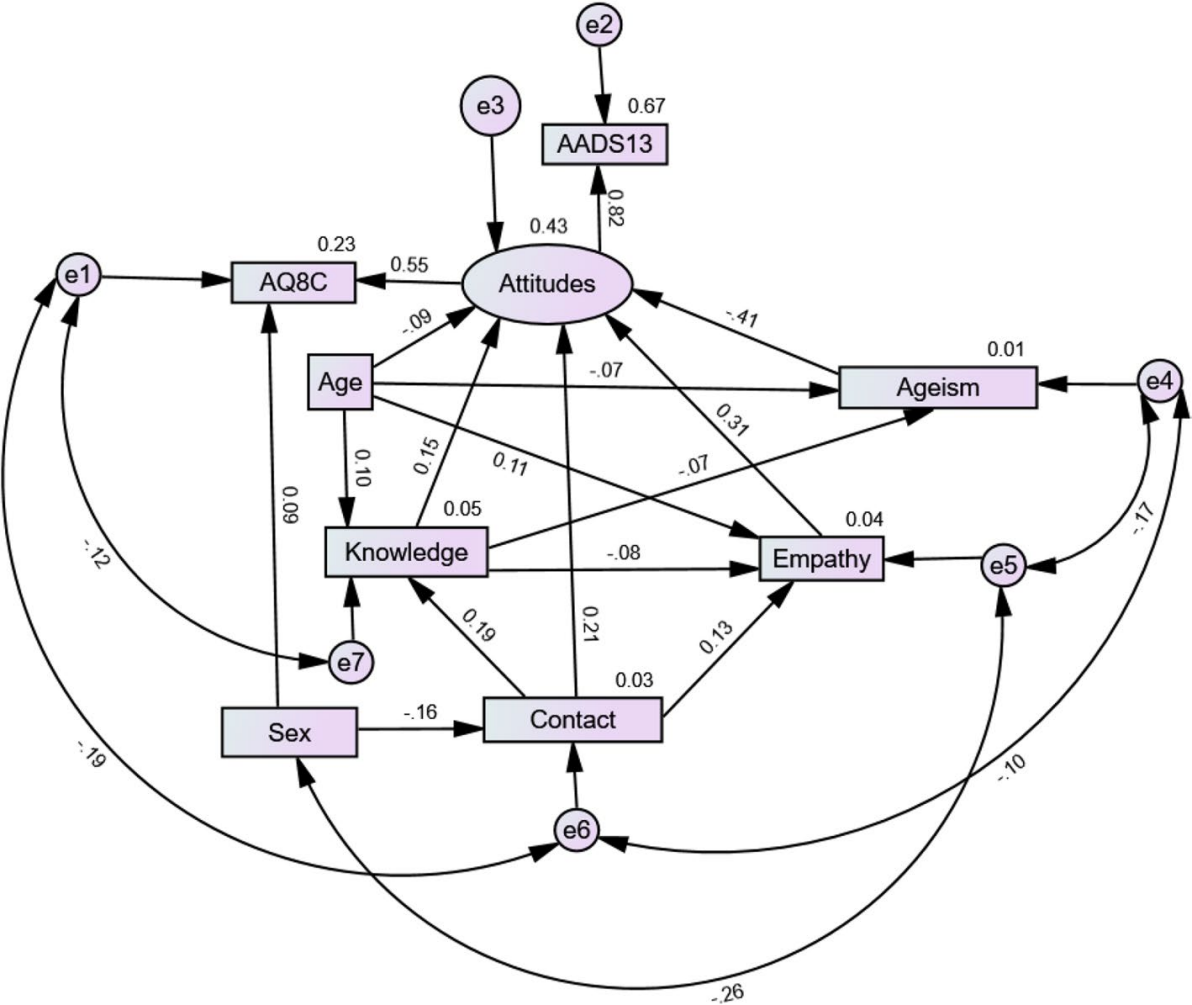
SEM model: accepted model with standardised coefficients

The SEM suggested the overall fit of the model was very good with several indicators of a very close fit, χ^2^(7) = 10.02* p* = 0.19, CMIN/DF = 1.43, CFI = 1.00, GFI = 1.00, AGFI = 0.99, TLI = 0.99, RMSEA = 0.02, Pclose = 0.97. The Bollen-Stine (*p* = 0.32) suggested that goodness of fit of the model was very good. Figure 1 displays the accepted model.

In the accepted SEM, all direct effects were statistically significant. The largest effects were contact (β = 0.21), empathy (β = 0.31), and ageism (β = -0.41). Table 3 reports the standardised coefficients and confidence intervals. All indirect pathways in the SEM were statistically significant. The strongest association was being male, and this was associated with less positive attitudes via the mediator contact (sex > contact > dementia-related stigma; β = -0.25, *p* = 0.00). Age also positively indirectly influenced dementia-related stigma via the mediator empathy (age > empathy > dementia-related stigma; β = 0.12 *p* = 0.00). Table 4 reports the standardised coefficients for the mediation effects.

**Table 3:** Direct effects of the accepted model

Parameters			β	B	S.E	Lower CI [95%]	Upper CI [95%]
Contact	←	Sex	-.16**	-2.42	.03	-.22	-.10
Knowledge	←	Age	.10**	.02	.03	.04	.16
Knowledge	←	Contact	.19**	.01	.03	.13	.25
Empathy	←	Age	.11**	.69	.03	.05	.17
Ageism	←	Age	-.07*	-.28	.03	-.14	-.01
Ageism	←	Knowledge	-.07*	-1.22	.03	-.13	-.01
Empathy	←	Knowledge	-.08*	-2.36	.03	-.14	-.01
Empathy	←	Contact	.13**	.11	.03	.07	.19
Dementia-related stigma	←	Age	-.09*	-.32	.04	-.16	-.02
Dementia-related stigma	←	Knowledge	.15**	2.68	.03	.09	.22
Dementia-related stigma	←	Empathy	.31**	.18	.04	.23	.39
Dementia-related stigma	←	Ageism	-.41**	-.41	.05	-.50	-.33
Dementia-related stigma	←	Contact	.21**	.10	.04	.13	.29

If 0 falls between the lower and upper bound, the effect is not statistically significant. Estimates with (-) = negative relationship. ‘Attitudes’ as a latent variable in the SEM is the outcome named ‘dementia-related stigma’

*β* Standardised beta regression coefficient. *Estimate (B)* unstandardised beta regression coefficient, *S.E* standard error of the standardised regression weight estimate, *β CI* standardised bias-corrected (95%) confidence interval (lower and upper bound)

Statistical significance: * = *p* < 0.05; ** = *p* < 0.01

**Table 4:** Mediation effects of the accepted model

Parameters	β	S.E	Lower CI [95%]	Upper CI [95%]
Contact > empathy > dementia-related stigma	.02**	.01	.01	.03
Sex > contact > dementia-related stigma	-.25**	.07	-.42	-.13
Contact > knowledge > dementia-related stigma	.01**	.00	.01	.02
Age > knowledge > dementia-related stigma	.06**	.02	.02	.11
Contact > knowledge > ageism	-.01*	.00	-.01	-.00
Age > empathy > dementia-related stigma	.12**	.04	.06	.22
Sex > contact > knowledge > ageism	.02*	.01	.00	.04
Sex > contact > knowledge > dementia-related stigma	-.03**	.01	-.07	-.02
Sex > contact > knowledge	-.01**	.00	-.02	-.01

If 0 falls between the lower and upper bound, the indirect effect is not statistically significant. Estimates with (-) = negative relationship. ‘Attitudes’ as a latent variable in the SEM is the outcome named ‘dementia-related stigma’

*β* standardised regression coefficient estimate, *S.E* standard error of the regression weight estimate. *β CI* bias corrected [95%] confidence interval (lower and upper bound)

Two-tailed statistical significance: * = *p* < 0.05; ** = *p* < 0.01

## Discussion

This is the first study to explore factors associated with dementia-related stigma outcomes in a diverse sample of adolescents across England. The study highlights several mechanisms that influence dementia-related stigma, through certain modifiable factors including level of contact, ageism, and empathy.

In line with other quantitative [52, 53] and qualitative [24] studies, higher levels of contact were associated with reduced dementia-related stigma. Our findings support models that highlight contact as an effective strategy to tackle stigma more broadly [54] and may also be an important modifiable component within adolescents. Interestingly, our findings indicate that being male was associated with less contact with dementia (direct effect), which in turn had an indirect effect on dementia-related stigma (sex > contact > dementia-related stigma). As such, previously reported gender differences between dementia-related stigma [13], may be better explained by the fact that females are more likely to engage with people with dementia. Possible reasons for this include that females often take on caregiving roles for family members [55]. Females are also more likely to seek health information in general [56]. Our findings highlight that adolescents who display higher levels of empathy were associated with less dementia-related stigma. There has been a limited number of studies quantitatively exploring the association between empathy and dementia-related stigma in adolescents. It has previously been argued that a better understanding of the role of empathy is needed [25] with only one prior qualitative study highlighting a potential link [24]. Similarly, studies exploring empathy and dementia attitudes are limited in adults, where these have generally focused on healthcare professionals and in the context of caregiving [22, 57]. Theoretically, stimulating empathetic responses could reduce dementia-related stigma. Our findings perhaps indicate that general empathy is tied to empathy towards specific health conditions (e.g., mental illness). Yet, empathy is not static and can be developed in a range of ways [58]. Such an approach has been highlighted in the adolescent disability literature when developing contact interventions [59]. The current model does highlight an indirect effect between the level of contact and dementia-related stigma, which is mediated by empathy. The pathway is relatively small within the model, perhaps owing to the generic nature of the empathy outcome, which may not be tapped into by dementia contact. The larger indirect pathway between age and dementia-related stigma (mediated by empathy) supports the concept that there are developmental changes to empathy in adolescents [60].

Another key association was between ageism, specifically lower collective affinity to older adults, and dementia-related stigma. These findings support the limited existing evidence in adolescents by Werner and colleagues on the association between ageism and dementia-related stigma [53]. The idea that affinity towards older adults is associated with dementia-related stigma is perhaps unsurprising, as the majority of people with dementia are older adults. Dementia symptoms can often be incorrectly attributed to normal ageing or provide the foundation for common older adult stereotypes [61]. Such an interpretation is supported by the direct pathway between knowledge and the ageism variable. The demonstration that ageist attitudes are prevalent in adolescents and are associated with stigma, suggests that there is a need to actively tackle ageist misconceptions about dementia through incorporating anti-ageist features within anti-stigma interventions.

Despite our study comprising the most diverse sample of adolescents to date, we found no associations between ethnicity and dementia-related stigma. This differs from previous literature [52, 53]. Caution should be taken with the findings since White British participants were compared to all other ethnicities. Therefore, effects might be masked by variability between minority ethnic groups. In addition, disentangling culture and ethnicity can be difficult. It might be that culture is more pertinent. This could explain why some studies find an effect, due to clear cultural distinctions. Future work will need to further explore differences between individual ethnic (or cultural) groups.

There are some limitations to consider for this study. First, the cross-sectional nature of the study limits what causal inferences can be made about the data with statistical associations not necessarily equating to meaningful associations [62]. Following up with participants longitudinally could be one method to better enhance causal inferences. Second, the conceptual development of the Brief A-ADS and the AQ-8-C are different and therefore may capture different underlying factors. The AQ-8-C captures attributions (trait-like) and therefore lends itself to capturing stereotypes. This underlying conceptual difference may explain why despite AQ-8-C and Brief A-ADS correlating, the relationship was weak, leading to poor amalgamation of these indicators when devising a single latent construct for the SEM. It is therefore important to consider that concepts such as ‘dementia-related stigma’ or ‘public stigma’, whilst useful for generating narratives, are complex and composed of multiple constructs [2, 63]. However, it is a strength of this study to have included measures of dementia-related stigma that have been validated in adolescent populations that incorporates theoretical underpinning. Third, the low reliability scores observed in both the AQ-8-C and the NILTS may have impacted the consistency and accuracy of the results obtained from these measures. Finally, it is not clear the extent to which there is response bias in the cohort, as no demographic data was collected from schools and students who refused to participate.

## Conclusion

This study examined what factors are associated with dementia-related stigma in adolescents. Our findings highlight the modifiable factors such as level of contact, ageism, and empathy have a potentially important role in attitude formation towards dementia in adolescents. Thus, these factors may be useful targets for anti-stigma interventions. Stimulating empathetic responses through contact with people with dementia may be one such strategy to tackle dementia-related stigma in adolescents.

## Supplementary Information

Supplementary Material 1.

## Data Availability

The datasets generated during and/or analysed during the current study are not publicly available due ethical restrictions relating to underage participants. Data can be requested directly from the corresponding author upon reasonable request.
