# Supplementary material for: Factors associated with dementia-related stigma in British adolescents
Source: BMC Public Health. 2024 Oct 21;24:2896. doi: 10.1186/s12889-024-20419-7 (PMC11492476; doi:10.1186/s12889-024-20419-7)
Supplement: Supplementary file 1 — Supplementary Material 1. [file 12889_2024_20419_MOESM1_ESM.docx]

**Supplementary material A-E**

Supplementary material A – AQ-8-C vignette

Supplementary material B – Validity and internal consistency checks

Supplementary material B – Table S1 – Internal consistency of all measures

Supplementary material C – SEM measurement model

Supplementary material C – Figure S1: Measurement model

Supplementary material D – SEM methodology

Supplementary material E – Table S2 - Indicators of model fit

**Supplementary material A: AQ-8-C vignette**

Vignette: Charlie is an elderly friend of the family who will be moving into your home. Your parents explain that Charlie has dementia and was previously living in a care home.

**Supplementary material B – Validity and internal consistency checks**

*Social desirability*

Brief Social Desirability Scale (BSDS) [1] is a validated instrument measuring social desirability where there is a potential to respond is a socially desirable way to attitudinal based questionnaires. The measure includes four items with a two-point Likert scale ‘Yes’ and ‘No’. The BSDS does not over burden the attitudinal questions since other social desirability scales such as the Marlowe-Crowne Inventory have 33 items which extends the time significantly, which may contribute to boredom effects. An example question includes; ‘if you say to people that you will do something, do you always keep your promise no matter how inconvenient it might be?’ BSDS was dichotomized into low social desirability tendency (scores between 1-6) versus high social desirability tendency (6.1 and above) to assess levels of social desirability in the sample. 83.6% scored a low social desirability tendency (n = 873).

*Validity checks*

Item correlations with total scores for each measure was checked. All measures total scores correlated with all their respective items significantly: Brief AADS-13 (*p*<.001), ALOCD (*p*<.001) (indirect contact, *p*<.001; direct contact, *p*<.001), EmQue-Ca subscales (affective, *p*<.001; cognitive, *p*<.001 and, prosocial, *p*<.001), Ageism scale (*p*<.001), AQ-8-C (*p*<.001), and BSDS (*p*<.001). The NILTS total score correlated with five items of the NILTS (*p*<.05). Two items of the NILTS was not significantly correlated with the total score, “dementia is a mental illness” (*p* = .80) and “dementia is a normal of ageing” (*p* = .09).

There was a significant, negative correlation between the AADS13 and ageism (r_s_ = -.44, *p*<0.001). The BSDS and AADS13 were negligibly negatively correlated (r_s_ = -.26, *p*<0.001) as was the AQ-8-C and AADS13 (r_s_ = -.13, *p*<0.001). Ageism and BSDS were negligibly positively correlated (r = .24, *p*<0.001), as was the ageism scale and AQ-8-C (r = .08, *p*<.01). The BSDS and the AQ-8-C did not significantly correlate with each other (r = .04, *p* = .18). These findings are in line with the expectation of a significant but low strength of association between the measures. This demonstrates that these questionnaires measure their own unique constructs.

*Internal consistency*

The NILTS, AQ-8-C and BSDS demonstrated inadequate internal consistency. All other measures demonstrated good or excellent reliability. See supplementary material B table S1.

**Supplementary material B Table S1 – Internal consistency of all measures**

Table S1 – Internal consistency of all measures

| **Measures** | **Cronbach's Alpha** | **Cronbach's Alpha**  **(Standardized Items)** | **Items (n)** |
| --- | --- | --- | --- |
| **NILTS** | .38 | .39 | 7 |
| **Brief A-ADS** | .86 | .86 | 14 |
| **ALOCD Total** | .86 | .86 | 10 |
| **ALOCD (direct contact)** | .91 | .91 | 5 |
| **ALOCD (indirect contact)** | .74 | .74 | 5 |
| **Experience of Dementia** | .78 | .78 | 5 |
| **EmQue-Ca** | .88 | .88 | 18 |
| **Ageism** | .90 | .90 | 5 |
| **AQ-8-C** | .63 | .66 | 8 |
| **BSDS** | .40 | .39 | 4 |

**Note. Cronbach’s Alpha (α) of .70 or above indicates good – excellent internal consistency.**

**Supplementary material C – SEM measurement model**

The brief A-ADS and AQ-8-C had a weak correlation (despite significant *p* value), which highlights they measure their own unique constructs. In the initial SEM measurement model, the latent variable was not a good measure of the dementia-related stigma construct. This was because the indicators used to measure the latent variable (brief A-ADS and AQ-8-C) did not adequately capture the underlying construct. The weak correlation between the two indicators, may explain why the latent variable did not accurately capture the relationship between attitudes and stigma. Given the AQ-8-C had an alpha reliability of .62, the AQ-8-C underwent a principal component analysis (PCA) to reduce the items to improve its internal consistency. After removing the items indicated by the PCA, the AQ-8-C revealed a single factor loading with five items. The removal of the three items that did not load well increased the alpha to .82. With the revised AQ-8-C (consisting of the 5 items instead of 8), the latent variable captured dementia-related stigma. The brief A-ADS accounted for a greater amount of the variance in the attitude construct (likely due to the brief A-ADS consisting of 13 items versus 5 items from AQ-8-C).

Moreover, to create a latent variable where both variables scored in the same direction, the AQ-8-C was summed and underwent scale reversal so that higher scores equated to fewer stigmatizing attitudes, in line with the brief A-ADS. All items were checked and revealed a significant relationship between the latent variable and each item of the AQ-8-C and the brief A-ADS.

**Supplementary material C – Figure S1: Measurement model**


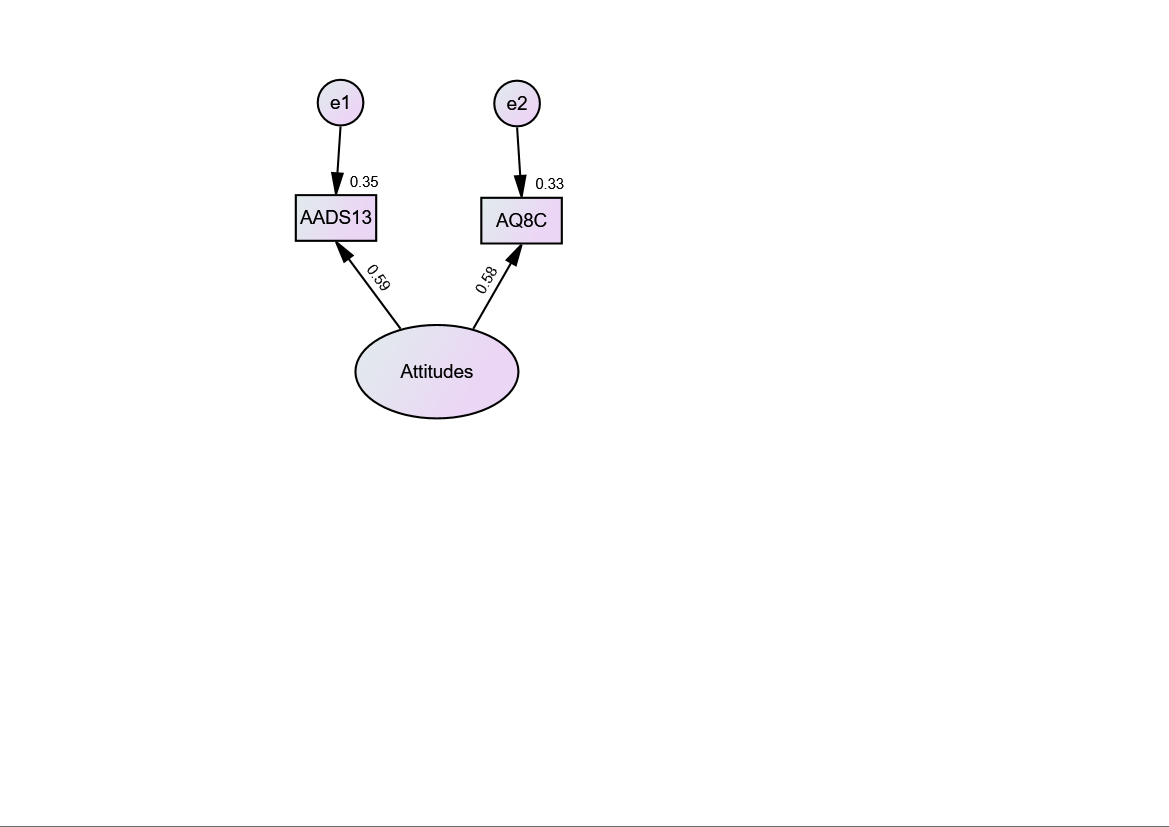


Figure S1 - SEM standardized estimates for accepted measurement model. Attitudes is the latent construct consisting of the brief A-ADS (13 items) and the AQ-8-C (5 items).

The measurement SEM model was recursive (n = 1044) (100,000 bootstraps) had an overall reasonable fit to the data, χ2(84) = 102.75, p = .08: AGFI = .98, GFI = .99, CMIN/DF = 1.22, RMSEA = .02, Pclose = 1.00, CFI = .99, and TLI = .99. Items between the brief A-ADS and the AQ-8-C were covaried where a correlation and suggested modification was indicated by the model. The Bollen-Stine revealed a good measurement model fit, *p* = .49. Factor loadings for brief A-ADS and AQ-8-C indicated by the maximum likelihood estimates was statistically significant (*p*<.001). In the accepted model, squared multiple correlations indicate that the latent variable accounts for 82% of the variance in brief A-ADS compared to 17% of the AQ-8-C.

**Supplementary material D – SEM methodology**

A bootstrapping procedure (100,000 samples) was applied due to having non-normal data [2]. Unstandardized coefficient betas, standardized coefficients, standard error (s.e.) and bias corrected CI (100,000 bootstrap samples at 95%) are reported for the direct and indirect effects for the CB-SEM [3] and to obtain more accurate estimations of the lower and upper limits of the CI [4]. A Bollen-Stine bootstrap was also utilised to obtain a goodness-of-fit statistic to provide the model fit (*p* value above 0.05 indicates good fit) [5].

**Supplementary material E Table S2 - Indicators of model fit**

Indexes and values used to judge whether the model fit is acceptable.

Table S2 - Indicators of model fit

| Indexes | Indication for acceptable model fit |
| --- | --- |
| Chi-square (χ2) *p* value | *p* ≥ .05 |
| Degrees of freedom (df) | > 3 |
| Goodness-of-fit index (GFI) | ≥ .90 |
| Adjusted goodness-of-fit index (AGFI) | ≥ .90 |
| Comparative fit index (CFI) | ≥ .90 |
| Tucker–Lewis index (TLI) | ≥ .90 |
| Root mean square error of approximation (RMSEA) | ≤ .05 (close fit) |
| Pclose | > .05 (close fit) |
| Bollen-Stine bootstrap – GFI | *p* > .05 |

***p* = p-value (statistical significance) [6,7]**

**Supplementary material references**

[1] Haghighat R. The development of the brief social desirability scale (BSDS). Europe’s Journal of Psychology. 2007 Nov 29;3(4):10-5964.

[2] Nevitt J, Hancock GR. Performance of bootstrapping approaches to model test statistics and parameter standard error estimation in structural equation modeling. Structural equation modeling. 2001 Jul 1;8(3):353-77.

[3] Cheung GW, Lau RS. Testing mediation and suppression effects of latent variables: Bootstrapping with structural equation models. Organizational research methods. 2008 Apr;11(2):296-325.

[4] Wagstaff DA, Elek E, Kulis S, Marsiglia F. Using a nonparametric bootstrap to obtain a confidence interval for Pearson’sr with cluster randomized data: a case study. The journal of primary prevention. 2009 Sep;30:497-512.

[5] Corrêa Ferraz R, Maydeu-Olivares A, Shi D. Asymptotic is better than Bollen-Stine bootstrapping to assess model fit: The effect of model size on the chi-square statistic. Structural Equation Modeling: A Multidisciplinary Journal. 2022 Sep 3;29(5):731-43.

[6] Bollen KA. Structural equations with latent variables. John Wiley & Sons; 1989 May 12.

[7] Resnick B, Galik E, Kolanowski A, VanHaitsma K, Boltz M, Zhu S, Ellis J, Behrens L, Eshraghi K, Renn C, Dorsey SG. The relationship between pain, function, behavioral, and psychological symptoms of dementia and quality of life. Pain Management Nursing. 2022 Feb 1;23(1):55-61.
